# Ticagrelor Resistance Causing Acute In-Stent Thrombosis: Successful Management With Prasugrel and Balloon Angioplasty

**DOI:** 10.14740/jmc5252

**Published:** 2026-02-02

**Authors:** Kaiyu Jia, Elizabeth Rimsky, Aysan Sattarzadeh, Arun Gajan Pradeep, Martin Miguel I. Amor

**Affiliations:** a Department of Medicine, Northwell Health at Staten Island University Hospital, Staten Island, NY 10305, USA; b Department of Cardiology, Northwell Health at Staten Island University Hospital, Staten Island, NY 10305, USA; c These authors contributed equally to this article.

**Keywords:** Percutaneous coronary intervention, In-stent thrombosis, Ticagrelor, Prasugrel, VerifyNow, Antiplatelet resistance

## Abstract

Ticagrelor is a cornerstone of dual antiplatelet therapy (DAPT) post-percutaneous coronary intervention (PCI), but resistance is rarely reported and poorly understood. We present a case of a 51-year-old woman with type 2 diabetes and a family history of coronary artery disease. Patient underwent elective PCI for severe proximal left anterior descending (LAD) and right coronary artery (RCA) stenoses, receiving aspirin and ticagrelor. Post-procedure, she developed syncope, hypotension, and ST-elevation on electrocardiogram (EKG), with repeat angiography revealing acute in-stent thrombosis in the proximal LAD. VerifyNow assay revealed ticagrelor resistance (307 PRU, repeat 293 PRU; cutoff < 208 PRU). Management included balloon angioplasty and transition to prasugrel (60 mg load, 10 mg daily). Angioplasty restored patency with no further events. Follow-up VerifyNow showed adequate inhibition (180 PRU) at 1 month, and the patient remained asymptomatic. Ticagrelor resistance can cause severe complications like in-stent thrombosis; VerifyNow-guided switch to prasugrel may prevent adverse outcomes, underscoring the need for tailored antiplatelet therapy. She underwent successful balloon angioplasty and was transitioned to prasugrel with no further events.

## Introduction

Ticagrelor, a reversible P2Y12 inhibitor, is a cornerstone of dual antiplatelet therapy (DAPT) and monotherapy following percutaneous coronary intervention (PCI) due to its rapid onset and more effective platelet inhibition compared to clopidogrel [1, 2]. While clopidogrel resistance is common (15–30%), ticagrelor resistance is rare, with reported rates of 0–20% [2–4]. Acute in-stent thrombosis, a life-threatening PCI complication, can result from antiplatelet resistance, leading to type 4b myocardial infarction. The mechanisms of ticagrelor resistance remain poorly defined, with no consensus on guidelines [2]. We report a case of acute in-stent thrombosis due to ticagrelor resistance, managed successfully with prasugrel transition and balloon angioplasty, and discuss diagnostic and therapeutic strategies.

## Case Report

**Figure 1:**
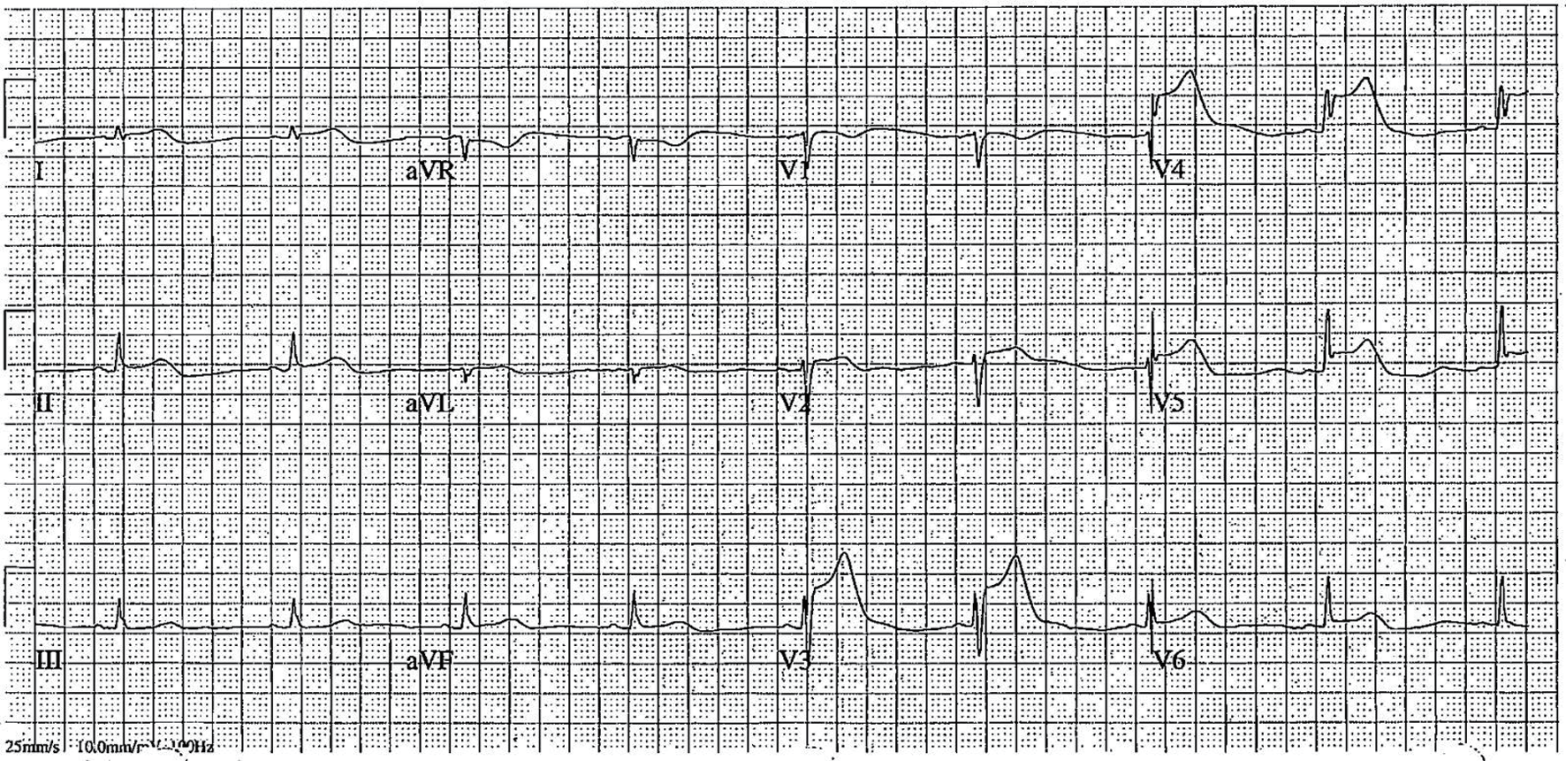
Electrocardiogram post-syncope showing ST-elevation in leads V3–V6, suggestive of acute in-stent thrombosis in the left anterior descending artery.

A 51-year-old woman with type 2 diabetes mellitus and a family history of coronary artery disease (CAD) presented for elective left heart catheterization (LHC). Outpatient coronary computed tomography angiography (CCTA) revealed severe proximal left anterior descending (LAD) stenosis, moderate multifocal right coronary artery (RCA) stenosis, and a coronary calcium score of 613. LHC confirmed 80% stenosis in the proximal LAD and proximal-to-mid RCA. Drug-eluting stents (SYNERGY 2.5 × 48 mm for LAD, 3.0 × 48 mm for RCA) were implanted following loading doses of aspirin (324 mg) and ticagrelor (180 mg) (Supplementary Videos 1 and 2, jmc.elmerpub.com). Within 2 h post-PCI, the patient developed syncope, hypotension, and ST-elevation in the inferior leads (Fig. 1). Differential diagnoses included acute in-stent thrombosis, stent malapposition, coronary artery dissection, or distal embolization. The patient was started on norepinephrine drip and returned to the catheterization lab.

**Figure 2:**
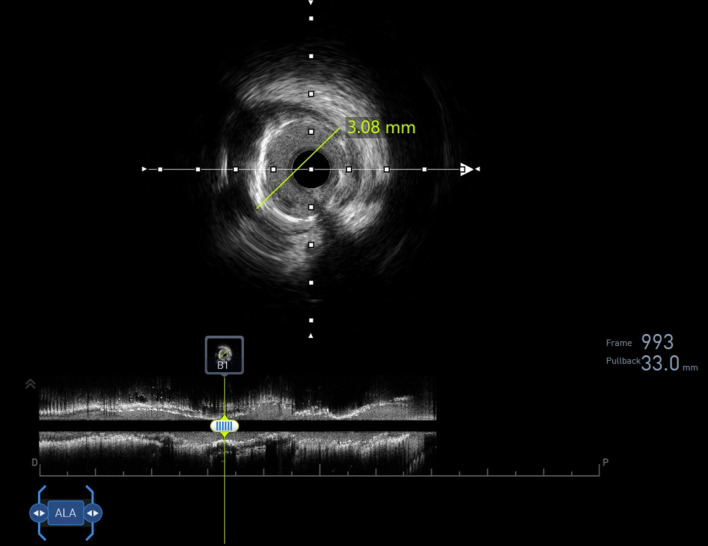
Intravascular ultrasound confirming 99% in-stent thrombosis in the proximal left anterior descending (LAD) stent, with minimal lumen area of 1.2 mm^2^.

Urgent repeat angiography revealed 99% in-stent thrombosis in the proximal LAD (Supplementary Video 3A, jmc.elmerpub.com), confirmed by intravascular ultrasound (IVUS) showing a minimal lumen area of 1.2 mm^2^ (Fig. 2). Balloon angioplasty (Boston Scientific 3.0 × 15 mm, 12 atm, 20 s) restored LAD stent patency (Supplementary Video 3B, C, jmc.elmerpub.com). The RCA stent remained patent (Supplementary Video 4, jmc.elmerpub.com). VerifyNow assay showed *in vitro* high residual platelet reactivity, suggesting ticagrelor resistance (307 PRU, repeat 293 PRU; cutoff < 208 PRU). Hypercoagulable workup (antithrombin III, protein S and C, anticardiolipin antibodies) was negative. A 60 mg prasugrel loading dose was administered, followed by heparin infusion (target activated partial thromboplastin time (aPTT) 53–73 s) for 17 h per acute coronary protocol. Norepinephrine was discontinued 1 h post-procedure. Post-procedural transthoracic echocardiography (TTE) showed a left ventricular ejection fraction of 62% with no thrombus.

The patient was monitored in the coronary care unit overnight and discharged on post-procedure day 1 with DAPT (aspirin 81 mg and prasugrel 10 mg daily) for 12 months. At 1-month follow-up, she remained asymptomatic with no recurrent cardiovascular events. Repeat VerifyNow assay confirmed adequate platelet inhibition (180 PRU). Diabetes management was optimized with metformin intensification, and lifestyle counseling was provided.

## Discussion

Ticagrelor is a first-line antiplatelet agent for CAD patients undergoing PCI, especially with complex coronary anatomy. According to the results of the PLATO randomized controlled trial, compared to clopidogrel, ticagrelor provided significant benefits in terms of reducing cardiovascular death, stroke, and myocardial infarctions in patients of non–ST-segment elevation acute coronary syndromes (NSTE-ACS) [5]. The superiority of ticagrelor was then echoed by multiple retrospective cohort studies later on [6–8].

Ticagrelor is a reversible, nonthienopyridine, P2Y12 inhibitor that binds to the target receptor in a non-adenosine diphosphate (ADP) competitive manner, which may attribute to its faster onset and more effective inhibitory ability on platelet aggregation [9]. Study have also demonstrated that compared to clopidogrel, patients who are on ticagrelor had reduced levels of plasma plasminogen activator inhibitor-1 (PAI-1), which is a key modulator of fibrinolysis, suggesting the additional profibrinolytic benefits of ticagrelor apart from platelet inhibition [10]. In contrast to the relatively higher reported prevalence of clopidogrel resistance of approximately 15–30% [11, 12], ticagrelor resistance is less common and has a reported prevalence ranging from 0% to 20% in various studies [2–4]. The mechanism of ticagrelor resistance remains unclear but is suspected to be multifactorial including CYP3A4 polymorphisms, drug interactions, or patient-specific factors like diabetes, though these were not tested in this case. Recent studies also found that the expression of P-glycoprotein on the intestinal epithelia is a contributing factor to ticagrelor absorption and efficacy [2]. There have also been studies that demonstrated the correlation between specific miRNAs with the expression of P2Y12 receptors in platelets [2]. Furthermore, another study has also shown that the use of morphine in STEMI patients was associated with delayed onset of action of ticagrelor [13]. Last but not least, medication non-adherence should also be considered when diagnosing ticagrelor resistance.

Currently, there are four ADP-stimulated assays available to test for resistance: VASP, Multiplate, VerifyNow, and light transmission aggregometry (LTA), with VerifyNow being the most widely used method. Studies have already proven that measurement of platelet response on P2Y12 inhibitor can be used as an independent predictor of recurrent cardiovascular events such as stent thrombosis. According to the GRAVITAS trial, a cutoff value of VerifyNow assay of less than 208 PRU is associated with lower risk of cardiovascular events [14]. In addition, the ADAPT-DES trial also demonstrated the validity of tailoring antiplatelet agents according to VerifyNow assay to prevent hemorrhagic and/or ischemic cardiovascular complications [15]. In our case, VerifyNow assay showed *in vitro* high residual platelet reactivity, suggesting partial or complete resistance to ticagrelor as the cause of this patient’s acute in-stent thrombosis immediately after the procedure, consistent with prior studies linking high platelet reactivity to stent thrombosis [14, 15].

In terms of the management of ticagrelor resistance, timely recognition and intervention are crucial in preventing serious cardiovascular complications. In our case, post-procedure close monitoring allowed for prompt recognition of the hemodynamic collapse. The use of IVUS was also critical in confirming thrombosis and ruling out malapposition. Compared to coronary angiography, IVUS offers a more detailed visualization of the culprit vessel and its intraluminal anatomy, stent position, plaque composition, and burden. The use of IVUS also plays an important role in reducing major adverse cardiac events (MACEs) and improving outcomes in PCI patients.

After ticagrelor resistance was detected, alternative P2Y12 inhibitors should be considered as part of DAPT. According to the TRITON-TIMI 38 trial, compared to clopidogrel, prasugrel was associated with significantly reduced rates of ischemic events, including stent thrombosis in ACS patients [16]. In addition, the PRASTRO-III study also demonstrated that prasugrel had comparable risks of bleeding events compared to clopidogrel [17]. In our case, prasugrel was chosen over clopidogrel due to its faster onset and lower resistance rates. A prasugrel loading dose was administered, along with a heparin drip, to prevent further stent thrombosis. The patient responded well to prasugrel with no further cardiovascular events.

This case highlights the importance of rapid recognition of resistance via VerifyNow and tailored antiplatelet therapy, suggesting that the testing of platelet function could be integrated as a part of the routine clinical practice to minimize ischemic events such as stent thrombosis. Limitations of this study include the lack of genetic testing and long-term follow-up data beyond 1 month.

### Conclusions

Ticagrelor resistance, though rare, can cause severe PCI complications like acute in-stent thrombosis. VerifyNow-guided transition to prasugrel effectively mitigates risks in this case, underscoring the need for personalized antiplatelet therapy post-PCI.

### Learning points

Recognize ticagrelor resistance as a rare but critical cause of in-stent thrombosis.

Understand the role of VerifyNow assay in guiding antiplatelet therapy.

Appreciate the efficacy of prasugrel as an alternative P2Y12 inhibitor in ticagrelor-resistant patients.

## Supplementary Material

Suppl 1 Angiography showing 80% stenosis in proximal LAD (A) and PCI with SYNERGY 2.5 × 48 mm at the stenosis site (B).

Suppl 2 Angiography showing 80% stenosis in proximal to middle RCA (A) and PCI with SYNERGY 3.0 × 48 mm at the stenosis site (B).

Suppl 3 Repeat angiography showing an acute in-stent thrombosis of 99% in proximal LAD (A), balloon angioplasty with Boston Scientific 3.0 × 15 mm balloon at the site of stent thrombosis (B) and subsequent successful revascularization (C).

Suppl 4 Repeat angiography showing patent stent in RCA.

## Data Availability

The authors declare that data supporting the findings of this study are available within the article.

## References

[1] Visseren FLJ, Mach F, Smulders YM, Carballo D, Koskinas KC, Back M, Benetos A (2021). 2021 ESC Guidelines on cardiovascular disease prevention in clinical practice. Eur Heart J.

[2] He S, Lin Y, Tan Q, Mao F, Chen K, Hao J, Le W (2023). Ticagrelor resistance in cardiovascular disease and ischemic stroke. J Clin Med.

[3] Wang Y, Chen W, Lin Y, Meng X, Chen G, Wang Z, Wu J (2019). Ticagrelor plus aspirin versus clopidogrel plus aspirin for platelet reactivity in patients with minor stroke or transient ischaemic attack: open label, blinded endpoint, randomised controlled phase II trial. BMJ.

[4] Choi WG, Kim GC, Lee CH, Kim HY, Kim DW (2021). The effect of antiplatelet drug on coronary endothelial and microvascular function: comparison with ticagrelor and clopidogrel. Korean J Intern Med.

[5] Pollack CV, Davoudi F, Diercks DB, Becker RC, James SK, Lim ST, Schulte PJ (2017). Relative efficacy and safety of ticagelor vs clopidogrel as a function of time to invasive management in non-ST-segment elevation acute coronary syndrome in the PLATO trial. Clin Cardiol.

[6] Li J, Qiu H, Yan L, Guo T, Wang Y, Li Y, Zheng J (2021). Efficacy and safety of ticagrelor and clopidogrel in patients with stable coronary artery disease undergoing percutaneous coronary intervention. J Atheroscler Thromb.

[7] Zheng W, Li Y, Tian J, Li L, Xie L, Mao Q, Tong W (2019). Effects of ticagrelor versus clopidogrel in patients with coronary bifurcation lesions undergoing percutaneous coronary intervention. Biomed Res Int.

[8] Li C, Liu M, Chen W, Jiang T, Ling L (2022). Comparison of ticagrelor and clopidogrel on platelet function and prognosis in unstable angina. Eur J Clin Pharmacol.

[9] Gragnano F, Mehran R, Branca M, Franzone A, Baber U, Jang Y, Kimura T (2023). P2Y(12) inhibitor monotherapy or dual antiplatelet therapy after complex percutaneous coronary interventions. J Am Coll Cardiol.

[10] Paszek E, Natorska J, Zabczyk M, Klajmon A, Undas A (2023). Therapy with ticagrelor/prasugrel is associated with enhanced fibrinolysis and suppressed platelet activation as compared to clopidogrel in chronic coronary syndrome. Kardiol Pol.

[11] Patrono C, Baigent C, Hirsh J, Roth G (2008). Antiplatelet drugs: American College of Chest Physicians Evidence-Based Clinical Practice Guidelines (8th Edition). Chest.

[12] Kuliczkowski W, Witkowski A, Polonski L, Watala C, Filipiak K, Budaj A, Golanski J (2009). Interindividual variability in the response to oral antiplatelet drugs: a position paper of the Working Group on antiplatelet drugs resistance appointed by the Section of Cardiovascular Interventions of the Polish Cardiac Society, endorsed by the Working Group on Thrombosis of the European Society of Cardiology. Eur Heart J.

[13] Parodi G, Bellandi B, Xanthopoulou I, Capranzano P, Capodanno D, Valenti R, Stavrou K (2015). Morphine is associated with a delayed activity of oral antiplatelet agents in patients with ST-elevation acute myocardial infarction undergoing primary percutaneous coronary intervention. Circ Cardiovasc Interv.

[14] Price MJ, Angiolillo DJ, Teirstein PS, Lillie E, Manoukian SV, Berger PB, Tanguay JF (2011). Platelet reactivity and cardiovascular outcomes after percutaneous coronary intervention: a time-dependent analysis of the Gauging Responsiveness with a VerifyNow P2Y12 assay: Impact on Thrombosis and Safety (GRAVITAS) trial. Circulation.

[15] Stone GW, Witzenbichler B, Weisz G, Rinaldi MJ, Neumann FJ, Metzger DC, Henry TD (2013). Platelet reactivity and clinical outcomes after coronary artery implantation of drug-eluting stents (ADAPT-DES): a prospective multicentre registry study. Lancet.

[16] Wiviott SD, Braunwald E, McCabe CH, Montalescot G, Ruzyllo W, Gottlieb S, Neumann FJ (2007). Prasugrel versus clopidogrel in patients with acute coronary syndromes. N Engl J Med.

[17] Kitazono T, Kamouchi M, Matsumaru Y, Nakamura M, Umemura K, Matsuo H, Koyama N (2023). Efficacy and safety of prasugrel vs clopidogrel in thrombotic stroke patients with risk factors for ischemic stroke recurrence: a double-blind, phase III study (PRASTRO-III). J Atheroscler Thromb.

